# Expression of Trefoil Factor 1 in the Developing and Adult Rat Ventral Mesencephalon

**DOI:** 10.1371/journal.pone.0076592

**Published:** 2013-10-07

**Authors:** Pia Jensen, Michel Heimberg, Angelique D. Ducray, Hans R. Widmer, Morten Meyer

**Affiliations:** 1 Department of Neurobiology Research, Institute of Molecular Medicine, University of Southern Denmark, Odense, Denmark; 2 Department of Neurosurgery, University of Bern, Bern, Switzerland; INSERM / CNRS, France

## Abstract

Trefoil factor 1 (TFF1) belongs to a family of secreted peptides with a characteristic tree-looped trefoil structure. TFFs are mainly expressed in the gastrointestinal tract where they play a critical role in the function of the mucosal barrier. TFF1 has been suggested as a neuropeptide, but not much is known about its expression and function in the central nervous system. We investigated the expression of TFF1 in the developing and adult rat midbrain. In the adult ventral mesencephalon, TFF1-immunoreactive (-ir) cells were predominantly found in the substantia nigra pars compacta (SNc), the ventral tegmental area (VTA) and in periaqueductal areas. While around 90% of the TFF1-ir cells in the SNc co-expressed tyrosine hydroxylase (TH), only a subpopulation of the TH-ir neurons expressed TFF1. Some TFF1-ir cells in the SNc co-expressed the calcium-binding proteins calbindin or calretinin and nearly all were NeuN-ir confirming a neuronal phenotype, which was supported by lack of co-localization with the astroglial marker glial fibrillary acidic protein (GFAP). Interestingly, at postnatal (P) day 7 and P14, a significantly higher proportion of TH-ir neurons in the SNc co-expressed TFF1 as compared to P21. In contrast, the proportion of TFF1-ir cells expressing TH remained unchanged during postnatal development. Furthermore, significantly more TH-ir neurons expressed TFF1 in the SNc, compared to the VTA at all four time-points investigated. Injection of the tracer fluorogold into the striatum of adult rats resulted in retrograde labeling of several TFF1 expressing cells in the SNc showing that a significant fraction of the TFF1-ir cells were projection neurons. This was also reflected by unilateral loss of TFF1-ir cells in SNc of 6-hydroxylase-lesioned hemiparkinsonian rats. In conclusion, we show for the first time that distinct subpopulations of midbrain dopaminergic neurons express TFF1, and that this expression pattern is altered in a rat model of Parkinson’s disease.

## Introduction

Idiopathic Parkinson’s disease (PD) is a common neurodegenerative disorder characterized by progressive loss of dopaminergic neurons in substantia nigra pars compacta (SNc) within the ventral mesencephalon. The resulting dopamine depletion leads to an imbalance in the nigrostriatal circuitry causing serious motor dysfunction [1,2]. Cardinal motor symptoms include resting tremor, muscle rigidity, bradykinesia, akinesia, and postural instability. However, non-motor symptoms are also present, these include depression, dementia, sleep disturbances and autonomic dysfunction [3]. Despite major efforts, the cause of the selective loss of dopaminergic neurons remains largely unknown, although several mechanisms have been proposed including oxidative stress, mitochondrial dysfunction and, excitotoxic damage [4]. Thus, more knowledge on the developmental biology of midbrain dopaminergic neurons, as well as molecules and mechanisms potentially taking part in the pathophysiology of PD, is clearly needed.

Midbrain dopaminergic neurons represent a heterogeneous cell population that can be divided into three principal groups; those of the the retrorubral area, SNc and the ventral tegmental area (VTA), also classified as the A8, A9 and A10 cell groups, respectively [5]. Besides being present in distinct locations, these dopaminergic neurons display characteristic projections. The A9 neurons of the SNc primarily innervate the dorsal striatum generating the nigrostriatal pathway, which is affected in PD. The A10 cell group mainly innervate ventral striatal and limbic areas as well as the frontal cortex and are relatively spared in PD, whereas the A8 cell group appears mainly to be involved in the interconnection of the SN and VTA [6,7].

Various criteria have been used to identify subtypes of neurons within these cell groups, including morphological differences and differences expression of the calcium-binding proteins calbindin (CB), calretinin (CR) and parvalbumin (PV) [8–11]. The SNc and VTA can thus be divided into subcompartments based on the distribution of dopaminergic cell populations expressing CR and/or CB. PV is mostly restricted to the ventral SN pars reticulata, which contains predominantly GABAergic neurons [10,12]. CR and CB show a distinct pattern of expression during development. The CR-immunoreactive (-ir) cells appear relatively early in the rostral SN, and are thereafter distributed throughout the SNc and VTA - most prominently in the ventral tier. CB-ir cells develop later and are particularly abundant in the VTA and dorsal tier of the SNc [13]. The functional significance of this compartmentalization remains unknown. Calcium-binding proteins are believed to regulate cellular activities such as cell proliferation, migration, and differentiation by suppressing or buffering intracellular calcium [14,15]. Notably, it has been shown that both CB-ir and CR-ir cells are more resistant to damage caused by PD and toxic insults than other nigral cells [16–19].

Trefoil factor 1 (TFF1) belongs to a peptide family (TFF1-3) most distinctly expressed in the gastrointestinal tract, where the peptides play essential roles in the function of the mucosal protective barrier. TFF peptides contain one or more characteristic trefoil domains, defined as a sequence of 42 or 43 amino acid residues in which 6 cysteines are disulphide-linked and form a characteristic three-leaved structure [20]. TFF peptides have been shown to influence precursor cell migration and epithelial restitution in the gastrointestinal tract and to protect cells against apoptosis [21]. All TFF peptides are synthesized via precursors containing a cleavable N-terminal sequence characteristic of secretory proteins [22]. Whilst no TFF receptors have yet been identified, receptor-ligand modes of action have been suggested [23,24]. TFF1 was first discovered in a human breast cancer cell line [25] and later shown to stimulate migration of breast cancer cells [26].

Several studies have shown that TFFs act as neuropeptides expressed in certain areas of the central nervous system (CNS), but the specific cellular expression and function of TFF1 remain largely unknown. In 1995, Hirota et al. reported that TFF1 mRNA was strongly expressed in rat hippocampus and moderately expressed in frontal cortex and cerebellum. Hippocampal TFF1 synthesis was found to peak around birth and then gradually decline from postnatal (P) day 7. Moreover, astrocytes were reported to represent the major site of TFF1 mRNA synthesis [27]. In accordance, expression of TFF1 mRNA in cultured mouse astrocytes has been found in the late G1 or early S phase under regulation of cytokines, such as IL-6, IL-7, and TNF-α [28,29]. However, these studies only address TFF1 mRNA levels by semi-quantitative PCR and have never been followed by more thorough data showing protein expression levels. Moreover, Hinz et al. analyzed the mRNA expression of TFF peptides in the mouse brain and found that TFF1 was expressed in many brain regions to varying extents, while TFF2 was predominantly found in the anterior pituitary. In contrast, TFF3 expression was limited to the hippocampus, the temporal cortex and the cerebellum, and TFF3 mRNA appeared to be restricted mainly to neurons [30]. Interestingly, Kriks et al. recently found highly enriched TFF3 mRNA levels in human embryonic stem cell cultures committed towards midbrain dopaminergic neurons as compared to cultures stimulated in a forebrain direction [31]. Any functional significance of this finding remains to be elucidated.

Tools to identify and distinguish subpopulations of midbrain dopaminergic neurons may provide new information on the specific cells that degenerate in PD and hence facilitate our understanding of the underlying disease mechanisms.

With the aim to test TFF1 as a potential supplementary marker of subsets of midbrain dopaminergic neurons we here investigated its expression in the developing and adult rat ventral mesencephalon by means of immunohistochemistry and retrograde tracing of nigrostriatal fibers. In addition, we studied the effects of unilateral, 6-hydroxydopamine (6-OHDA) lesions on the content and distribution of midbrain TFF1-ir cells.

## Experimental Procedures

### Animals and tissue processing

This study was carried out in strict accordance with the recommendations in the Law for the Care and Use of Laboratory Animals of the Swiss Authorities (BVET). The procedures were approved by the Animal Research Ethics Committee of the Canton Berne, Switzerland (Permit Numbers: 89/05, 6/12). All surgeries were performed under appropriate anaesthesia, and all efforts were made to minimize suffering of the animals (see below).

Under deep surgical anaesthesia (Ketamine 100mg/kg and Xylazine 10mg/kg) adult female Wistar rats (Janvier Elevage, France) were perfused through the ascending aorta using a perfusion pump, first with a prewash solution of 200 ml 0.1M PBS followed by 400 ml fixative (4% paraformaldehyde (PFA) in 0.1M PBS, pH 7.3). For that purpose the thorax was opened by a transdiaphragmal approach and heparin was administered intracardially (1000 I.E. /100 ml, Novo Nordisk, Denmark). To avoid perfusion of the whole body, the descending aorta was pinched off with a clamp. The rats were decapitated immediately after perfusion and the brains removed carefully from the skull. Post fixation was performed in 4% PFA/PBS (pH 7.3) for six hours and thereafter the brains were cryoprotected by immersion in PBS containing 10% sucrose and 0.01% NaN_3_. After freezing in isopentane at -80°C the brains were cut on a cryostat (Leica, AM1900) at 30 µm. The sections were mounted either directly on silane-coated superfrost microscope slides or stored in wells with anti-freeze solution (400 ml H_2_O, 300 ml glycerol, 300 ml ethylenglycol, 1.57 g NaH_2_PO_4_ and 5.18 g Na_2_HPO_4_) at -20°C. The brains were cut in series of six. Three sections were mounted on slides and three were stored as free-floating sections in wells. Each slide or well contained three sections that were 180 µm apart, thus covering a range of 360 µm.

Adult rat stomach tissue was collected for control immunohistochemical stainings. Briefly, the stomach was removed from deeply CO_2_-anaesthetized rats according to local and national animal ethics regulations. Stomachs were depleted from content by washing in D-PBS followed by immersion fixation in 4% PFA/PBS for six hours, and thereafter the tissue was cryoprotected in PBS containing 20% sucrose for at least 24 hours at 4°C. After, tissue was frozen using gaseous CO_2_ followed by cryostat sectioning at 30 µm and mounting on superfrost microscope slides.

### Unilateral 6-hydroxydopamine (6-OHDA)-lesions

Female Wistar rats (Janvier Elevage, France), weighing 220-250g were briefly sedated with isoflurane (75% N_2_O, 20% O_2_, 4.5-5% Isoflurane) before receiving an injection of Ketamine and Xylazine (Ketamine 75mg/kg and Xylazine 5mg/kg) and being placed in a stereotaxic frame (Kopf Instruments, USA). Prior to the incision, a subcutaneous injection of lidocaine HCl (1%) was administered. Striatal dopamine depletions were made by injection of 32 mM 6-OHDA hydrobromide (Sigma) in 4 µl saline supplemented with 0.02% ascorbic acid as an antioxidant into the right striatum through a small burrhole in the skull. The injection was performed over 6 min using a 10 µl Hamilton microsyringe. The injection rate was 1 µl/min. The syringe was left in place for an additional 4 min before removal [32]. The following coordinates in relation to Bregma were used: posterior 1.0 mm, lateral 3.0 mm and ventral -5.0 mm to the dura, the incisor bar was set at 0.0 mm [33]. Animals received a subcutaneous injection of Carprofen (5 mg/kg) as postoperative analgesic. After 4 weeks rats were perfused as described above.

### Fluorogold injection

Fluorogold is a reliable tracer for retrograde labelling of neurons [34]. Female Wistar rats (Janvier Elevage, France), weighing 220-250g were briefly sedated with isoflurane (75% N_2_O, 20% O_2_, 4.5-5% Isoflurane) followed by an injection of Ketamine and Xylazine (Ketamine 75mg/kg and Xylazine 5mg/kg) and placed in a stereotaxic frame (Kopf Instruments, USA). Prior to the incision, a subcutaneous injection of lidocaine HCl 1% was administered. 0.2 µl of 2% Fluorogold (Chemicon) in 0.9% NaCl was injected into the right striatum through a small burrhole created in the skull. Fluorogold was injected over 5 minutes using a 1 µl Hamilton needle. The needle was left in place for another 8 minutes and removed. The following coordinates in relation to Bregma were used: posterior 1.0 mm, lateral 3.0 mm and ventral -4.5 mm to the dura, the incisor bar was set at 0.0 mm [35]. Animals received a subcutaneous injection of Carprofen (5 mg/kg) as postoperative analgesic. After a survival time of 10 days, the animals were re-anesthetized and perfused as described above.

### Immunohistochemistry

Free-floating sections were washed four times in 0.1M PBS to remove the anti-freeze solution. Mounted sections were rinsed in PBS for 15 min. All sections were pre-incubated with PBS containing 0.4% Triton X-100 and 10% horse serum for 2 hours at room temperature. After a brief wash in PBS, sections were incubated with primary antibodies (rabbit anti-TFF1, 1:1000, Novocastra; rabbit anti-TH, 1:1000, Pelfreeze) diluted in PBS containing 0.1% Triton X-100 and 2.5% horse serum overnight at 4°C. Subsequently, sections were incubated with a secondary antibody solution consisting of biotinylated horse anti-rabbit or goat anti-mouse antibody (Vector laboratories) diluted 1:200 in PBS containing 0.1% Triton X-100 and 2.5% horse serum for 2 hours at room temperature. After washing for 4x10 min in PBS endogenous peroxidase was blocked by treating the sections with PBS containing 3.6% H_2_O_2_ and 10% methanol, followed by 3x10 min washes in PBS. Visualization of cell bound antibodies was performed using the avidin-peroxidase-complex system (Vector Laboratories) combined with a 3,3’-diaminobenzidine (DAB) substrate kit (Pierce). After 2x5 min wash in PBS the free-floating sections were mounted carefully on poly-L-lysine-coated slides with a brush and air-dried for an hour. All sections were dehydrated in graded ethanol (70%, 95%, 100%) for 3 min each, cleared in xylene and cover slipped with Eukitt mounting medium.

Negative control staining was performed by omitting either primary or secondary antibodies. Positive controls were performed by TFF1 immunostaining of rat stomach/intestinal tissue (Figure S1). To identify the optimal working concentration of the anti-TFF1 antibody, immunostaining was performed using dilutions of 1:250, 1:500 and 1:2000. Each dilution resulted in staining of cells in the SN (Figure S2). Notably, dilutions of 1:250 and 1:500 caused a rather strong background staining. In contrast, the 1:2000 dilution resulted in a weak background staining but also a weaker staining pattern. Hence, we decided to use a dilution of 1:1000 in all the following experiments.

To further verify our initial findings and evaluate antibody specificity, adjacent brain sections were immunostained using a monoclonal mouse anti-TFF1 antibody (anti-pS2, Clone BC04, Zymed Lab.) and the staining pattern compared with that of the antibody used in this study (rabbit polyclonal NCL-pS2, Novocastra, Leica Microsystems). Similar staining patterns were observed for the two antibodies as shown at the level of the subfornical organ and in the midbrain of adult rats (Figure S3). However, the monoclonal antibody resulted in a weaker staining pattern even after antigen retrieval. Unilateral lesion of TH-ir positive cells in the SNc of the 6-OHDA rat model of PD [32] resulted in partial depletion of TFF1-positive cells as detected by use of both antibodies (Figure S4).

Hence, the rabbit polyclonal anti-TFF1 was used throughout the present study.

### Immunofluorescence staining

Double immunofluorescence stainings were performed as described above with minor additions. Briefly, sections were incubated with a mixture of rabbit anti-TFF1 (1:500, Novocastra) and mouse anti-TH (1:1000, Chemicon), mouse anti-neuronal nuclei (NeuN) (1:100, Chemicon), mouse anti- glial fibrillary acidic protein (GFAP) (1:400, Chemicon), mouse anti-MAB1580 (1:1000, Chemicon), mouse anti-calbindin (CB) (1:2000, Swant), mouse anti-parvalbumin (PV) (1:2000, Swant) or mouse anti-calretinin (CR) (1:2000, Swant) diluted in PBS containing 0.1% Triton X-100 and 2.5% horse serum overnight at 4°C. Subsequently, sections were incubated with a mixture of Alexa Fluor® 488 conjugated donkey anti-mouse IgG and Alexa Fluor® 594 conjugated donkey anti-rabbit IgG (1:250, Molecular Probes) for 2 hours at room temperature. Cell nuclei were counterstained with Hoechst 33342 (Invitrogen, Molecular Probes) at 1:10,000. Finally, free-floating sections were mounted on poly-lysine-coated slides with a brush and air-dried for an hour in the dark. Sections were cover-slipped with PBS containing 50% glycerol (Merck) and examined using a digital camera (Nikon) equipped epifluorescence microscope (Leitz). Images were processed using Adobe® Photoshop® software.

### Cell counting and statistical analyses

Semi-quantitative analysis of midbrain TFF1-ir cells was performed on sections from 3 different control animals at the level: Bregma -5.8 mm. Sections were analyzed on a microscope (Olympus BX51) equipped with a digital camera (Olympus DP72). Pictures were taken at 100x magnification and stitched together to keep track of the brain areas and to have a sufficient resolution to distinguish single cells. A grid (200 µm x 200 µm) was overlaid, and TFF1-ir cells were counted in each square using the cell counting tool of CellF© (Olympus).

Quantitative analyses of TH and TFF1 co-localizing cells in the SN and VTA of P7, P14, P21 and adult rats were performed on fluorescent pictures taken at 100x magnification on a Leitz epifluorescence microscope equipped with a Nikon digital camera. The areas of SN and VTA were verified by the use of consecutive sections stained for TH using DAB as described above. Four to seven animals were used per time point with three to six brain sections used from each animal, and cell counts were performed on both sides of the brain. TH-ir and TFF1-ir cells were counted on single immunofluorescence pictures and subsequently the co-localizing cells were counted on pictures of double immunofluorescence-stained sections using Adobe® Photoshop® software.

Statistical analyses of cell densities were performed by means of commercially available software (Instat, GraphPad Software). Relative contents of TH/TFF1 or TFF1/TH co-expressing cells during development were compared using parametric one-way analysis of variance (ANOVA) followed by Student-Newman-Keuls multiple comparisons. The ratios of TH-ir cells expressing TFF1 and of TFF1-ir cells expressing TH in SN and VTA in adult rats were compared using a two-sided Fisher’s exact test. Differences were considered statistically significant at P<0.05. Values are presented as mean ± SEM.

## Results

### Expression of TFF1 in the adult rat ventral mesencephalon

**Figure 1:**
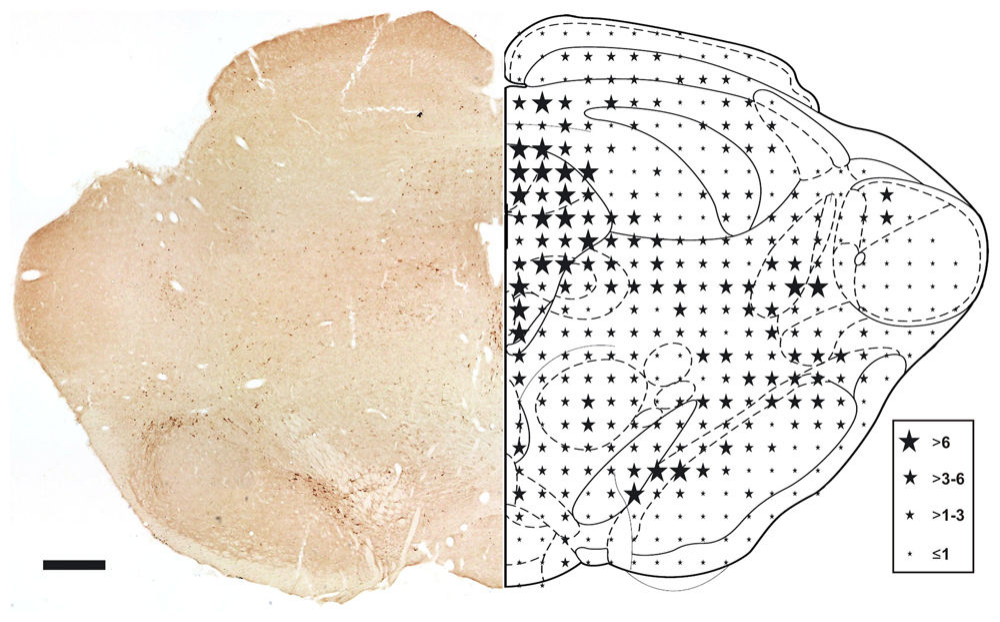
Trefoil factor 1 (TFF1) expression in the midbrain of adult rats. Representative photomicrograph depicting the distribution of TFF1-immunoreactive (-ir) cells in the midbrain (level: Bregma -5.8 mm). The corresponding schematic drawing shows the semi-quantitative representation of TFF1-ir cells in the midbrain. Scale bar: 1 mm.

**Figure 2:**
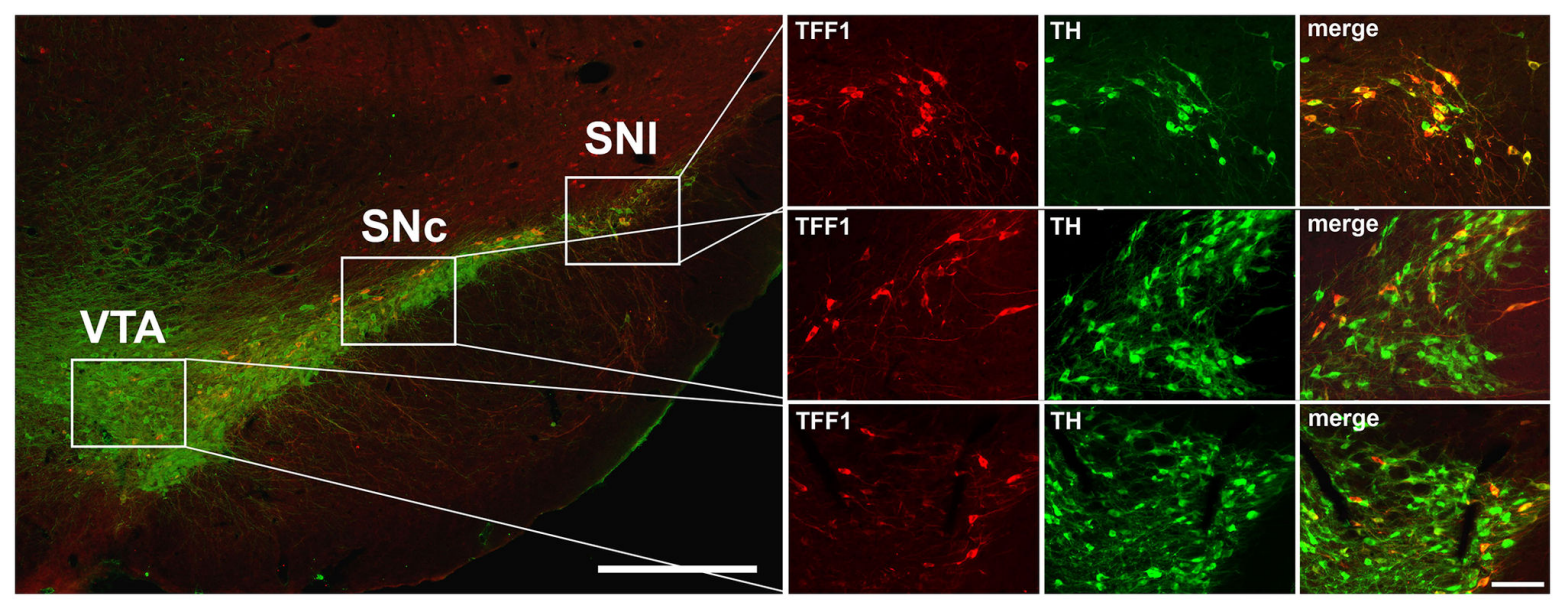
Co-expression of trefoil factor 1 (TFF1) and tyrosine hydroxylase (TH) in the ventral mesencephalon of adult rats. Double immunofluorescence staining for TFF1 (red) and TH (green) in the ventral mesencephalon of adult rats. Note that nearly all TFF1-immunoreactive (-ir) cells co-localized with TH, while many TH-ir cells did not co-express TFF1. Scale bars: 1 mm (overview); 100 µm (magnification). Abbreviations: SNc, substantia nigra pars compacta; SNl, substantia nigra pars lateralis; VTA, ventral tegmental area.

Immunohistochemical analyses of adult rat mesencephalic brain sections revealed that TFF1 was expressed throughout the midbrain, with the most pronounced expression in the substantia nigra (SN), the periaqueductal grey matter and the Edinger-Westphal nucleus. The distribution pattern of TFF1-ir cells in the midbrain is schematically summarized in Figure 1. In the ventral midbrain TFF1 was markedly expressed in cells of the SN pars compacta (SNc) and the SN pars lateralis (SNl), and to a lesser extent in the ventral tegmental area (VTA), while SN pars reticulata (SNr) contained almost no TFF1-ir cells (Figure 2).

**Figure 3:**
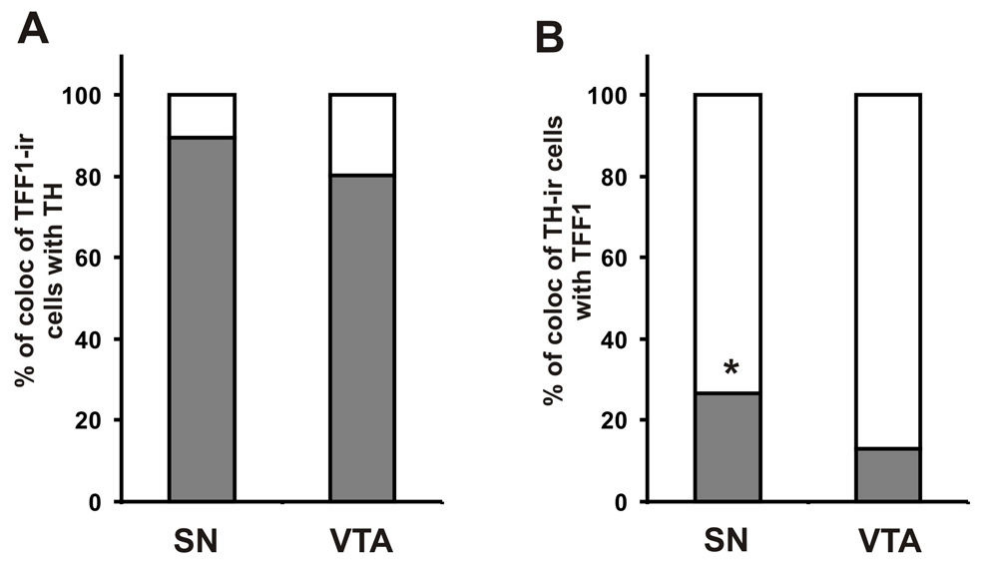
Quantification of trefoil factor 1 (TFF1) and tyrosine hydroxylase (TH) co-expressing cells in adult rats. Percentage of trefoil factor 1-immunoreactive (TFF1-ir) cells that co-localized with tyrosine hydroxylase (TH) (A) and percentage of TH-ir cells that co-localized with TFF1 (B) in the substantia nigra (SN) and ventral tegmental area (VTA) of adult rats. *: p<0.05 vs. corresponding VTA values, n = 4.

Quantification of the relative content of TFF1-ir cells that co-localized with TH revealed no significant difference between the SN and VTA (Figure 3A) (SN: 89.6 ± 0.9 and VTA: 80.2 ± 5.0% TFF1 co-localization with TH; mean ± SEM; n = 4), whereas the percentage of TH-ir cells that co-localized with TFF1 was significantly higher in the SN as compared to the VTA (Figure 3B) (SN: 26.8 ± 1.5 and VTA: 13.1 ± 1.2% TH co-localization with TFF1; mean ± SEM; n = 4; P<0.05).

Antibody specificity was verified by use of various positive and negative controls as well as another anti-TFF1 antibody confirming the observations described above (see Experimental procedures and Figure S1-4).

All experiments indicated that the anti-TFF1 antibody (Novocastra) was specific. Moreover, our data were supported by in silico data (Allen Brain Atlas).

### Co-localization of TH and TFF1-positive cells in the developing ventral mesencephalon

**Figure 4:**
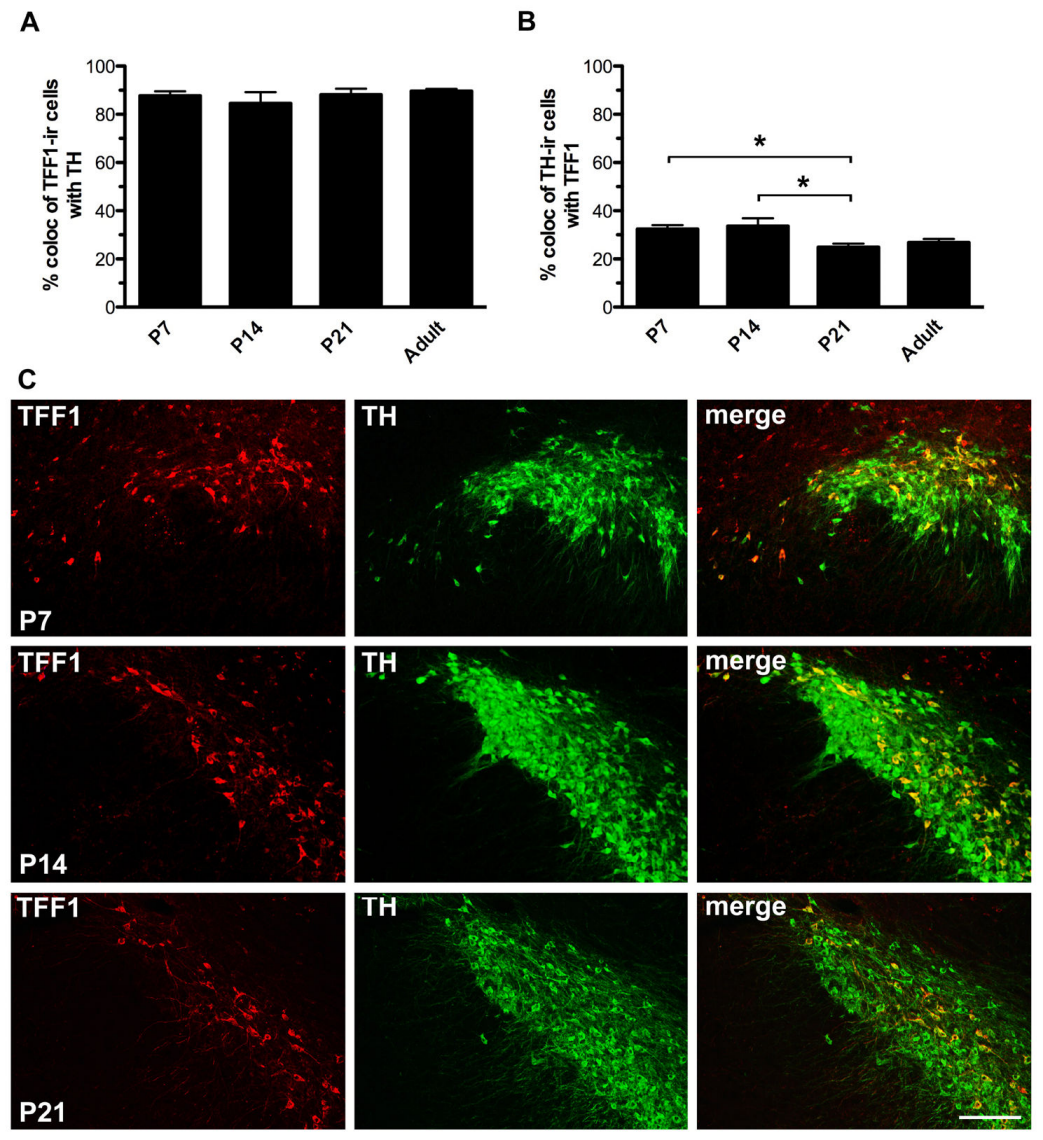
Quantification of trefoil factor 1 (TFF1) and tyrosine hydroxylase (TH) co-expressing cells during development. A) Quantification of trefoil factor 1-immunoreactive (TFF1-ir) cells co-expressing tyrosine hydroxylase (TH) and B) TH-ir cells co-expressing TFF1 in the substantia nigra (SN) of postnatal (P) day 7, 14, 21 and adult rats. The percentage of TH/TFF1 co-expressing cells was significantly higher for P7 and P14 rats compared to P21 rats, whereas there was no significant difference between the percentages of TFF1-ir cells co-expressing TH. Data are expressed as mean ± SEM (*: p<0.05, n = 4-7). C) Representative double immunofluorescence images of TFF1-ir and TH-ir cells in SN of P7, P14 and P21 rats (see Figure 2 for adult rats). Scale bar: 200 µm.

To investigate the expression of TFF1 in the SN and VTA during development we analysed brains from newborn (P0) as well as post-natal days P7, P14, P21 and adult rats. P0 rats had very few TFF1-ir cells in the SN/VTA (data not shown), while numerous TFF1-ir cells were seen in the SN (Figure 4C) and VTA (not shown) at the other time points. Quantification of TH-ir cells co-expressing TFF1 in the SN (referring to both SNc and SNl) revealed that the percentage of TH/TFF1 co-localization was significantly higher in P7 and P14 rats compared to P21, whereas there was no significant difference between P7, P14 and adult or P21 and adult rats (Figure 4B) (P7: 32.4 ± 1.6, P14: 33.6 ± 3.3, P21: 24.9 ± 1.5, adult: 26.8 ± 1.5% TH co-localization with TFF1; mean ± SEM; n = 4-7; P<0.05). In addition, there was no significant difference between the percentages of TFF1-ir cells co-expressing TH at any of the time points (Figure 4A) (P7: 87.7 ± 1.8, P14: 84.5 ± 4.7, P21: 88.1 ± 2.5, adult: 89.6 ± 0.9% TFF1 co-localization with TH; mean ± SEM; n = 4-7).

### Phenotypical characterization of TFF1-positive cells in the ventral mesencephalon

**Figure 5:**
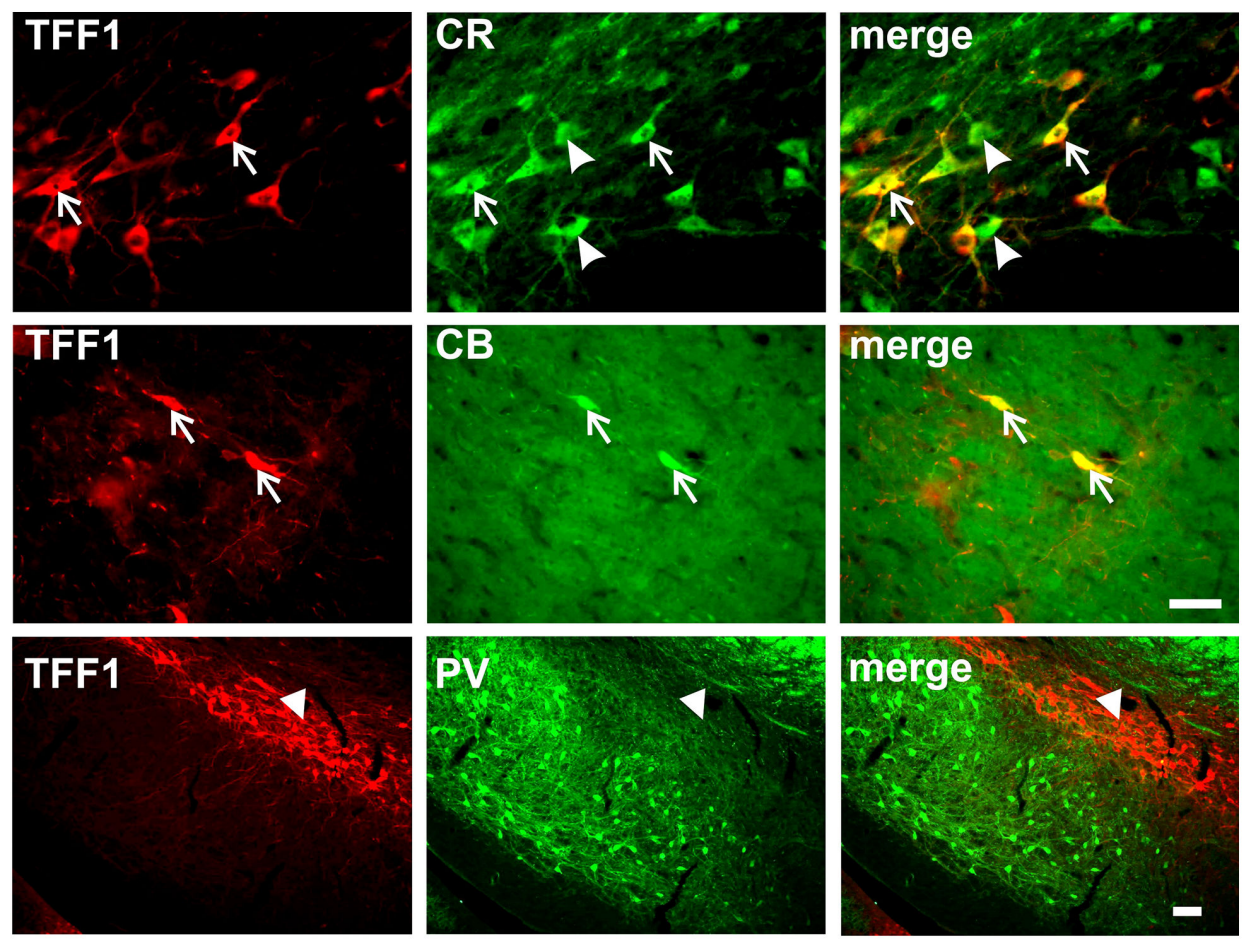
Co-expression of trefoil factor 1 (TFF1) and calcium-binding proteins in the ventral mesencephalon of adult rats. Double immunofluorescence stainings for TFF1 and the calcium-binding proteins calretinin (CR), calbindin (CB) or parvalbumin (PV). Note that many TFF1-immunoreactive (-ir) cells co-expressed CR (arrows), but not all CR-ir cells co-localized with TFF1 (arrowheads). Similarly, co-localization with CB was seen for a few TFF1-ir cells (arrows). As expected from the distribution pattern depicted in Figure 1 no co-localization was detected for TFF1-ir cells with PV (arrows). Scale bars middle panel: 50 µm, lower panel: 100 µm.

By double immunofluorescence staining we demonstrated that a high number of the TFF1-ir cells in the SNc, SNl and VTA co-localized with TH, however, only a subgroup of TH-ir neurons expressed TFF1 (Figures 2 and 3). Furthermore, a high number of the TFF1-ir cells co-expressed the calcium-binding protein calretinin (CR) but few co-expressed calbindin (CB). As expected by the outcome seen for TFF1 distribution in the SNr no co-localization was detected for TFF1 and parvalbumin (PV) (Figure 5).

**Figure 6:**
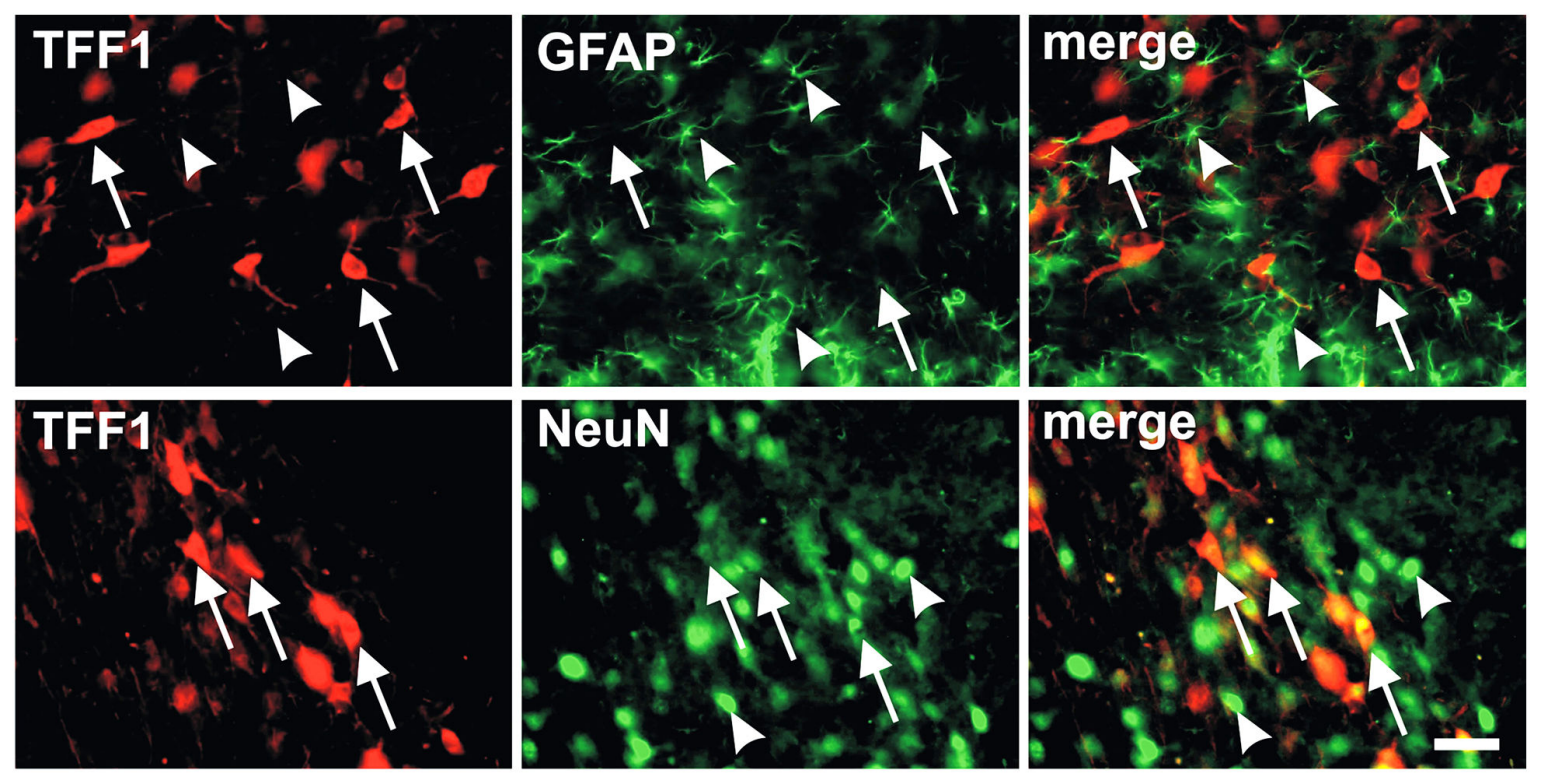
Trefoil factor 1 (TFF1) expression is restricted to neurons. Representative photomicrographs of double immunofluorescence stainings for TFF1 and astroglial or neuronal markers in the substantia nigra pars compacta (SNc) of adult rats. No co-localization was found of TFF1-positive cells (arrows) with the astroglial marker glial fibrillary acid protein (GFAP) (arrowheads). Notably, a substantial number of the TFF1-ir cells were demonstrated to co-express the pan neuronal marker NeuN (arrows), while some did not (arrowheads). Scale bar: 50 µm.

Double immunofluorescence staining revealed no co-localization between TFF1 and the astroglial marker GFAP in the ventral mesencephalon, whereas most of the TFF1-positive cells co-expressed the pan neuronal marker NeuN (Figure 6).

**Figure 7:**
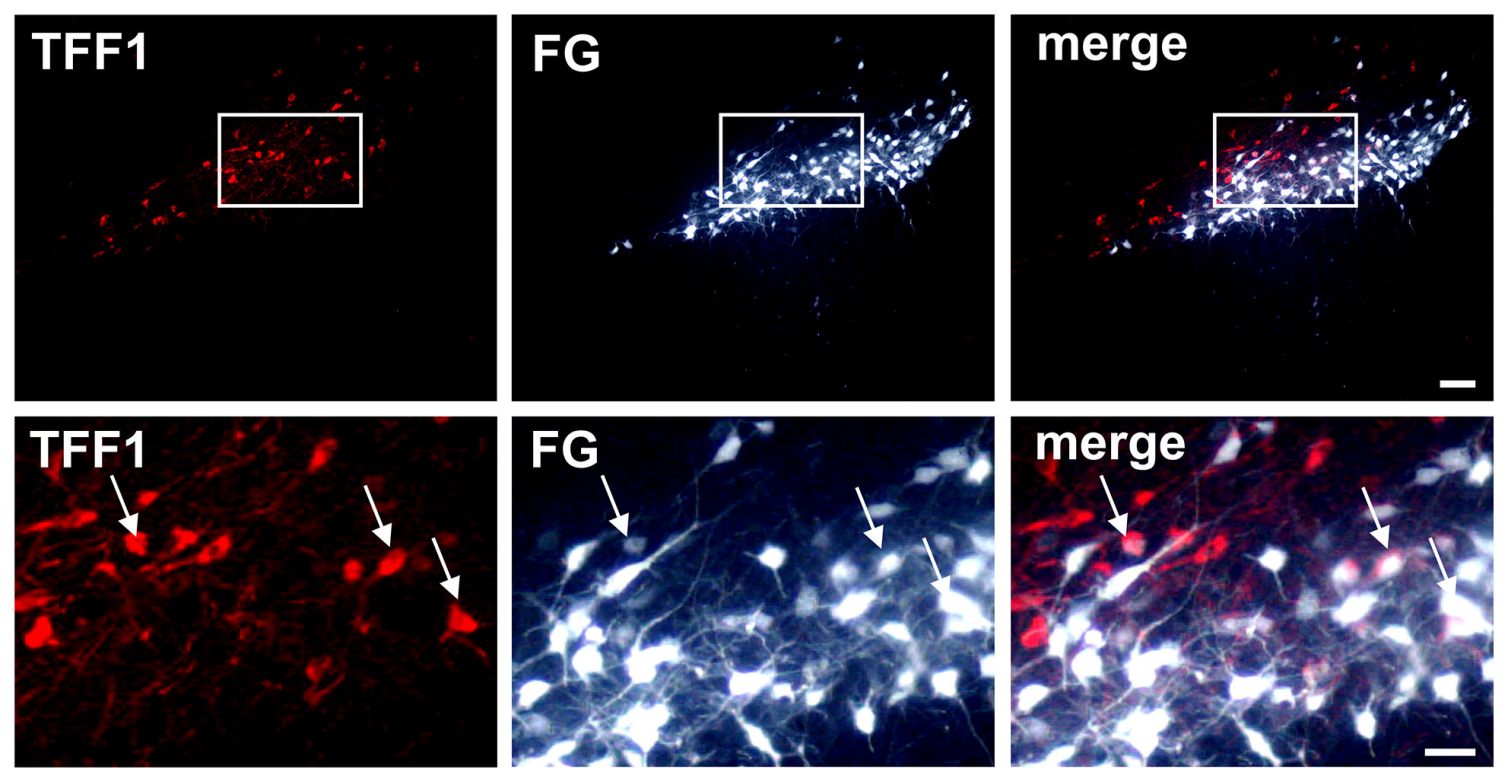
Detection of Trefoil factor 1 (TFF1) in nigrostriatal projection neurons by Fluorogold (FG) labelling. Representative photomicrographs of double immunofluorescence stainings for TFF1 and the retrograde tracer FG at the level of substantia nigra pars compacta (SNc) 10 days after intrastriatal FG injection. A subpopulation of TFF1-ir cells was found to co-express FG identifying these as projection neurons (arrows). Scale bars upper panel: 100 µm, lower panel: 50 µm.

**Figure 8:**
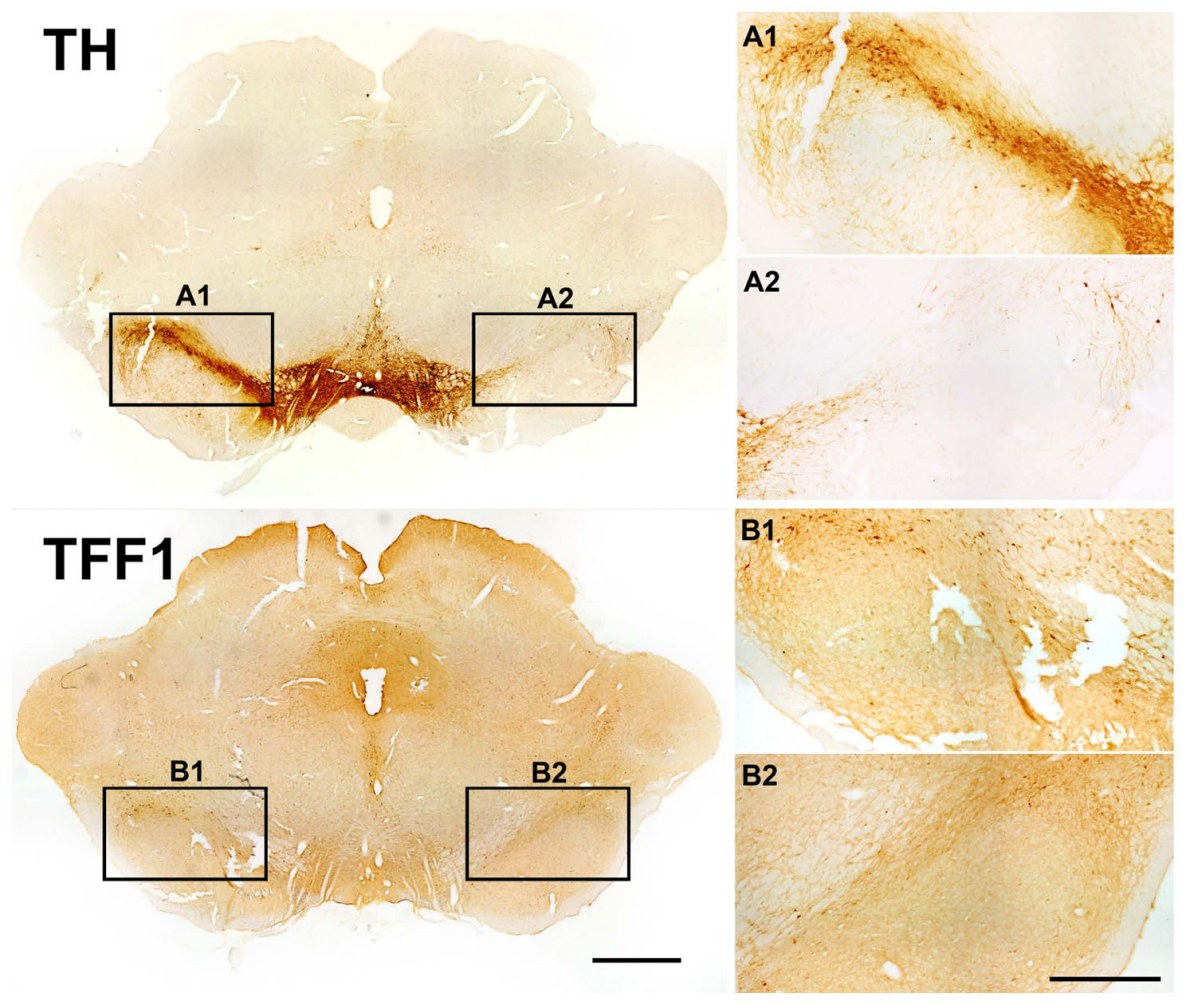
Loss of trefoil factor 1 (TFF1) expressing cells in a rat model of Parkinson’s disease. Representative photomicrographs of tyrosine hydroxylase (TH; A) and TFF1 (B) in brain sections from adult rat ventral mesencephalon at 4 weeks after unilateral 6-hydroxydopamine (6-OHDA) lesion. The lesion resulted in a distinct loss of TH-ir neurons in right SN (A2) as compared to the contralateral, unlesioned control side (A1). Similarly, a reduction of TFF1-ir cells was detected on the lesioned side (B2) as compared to the intact control side (B1). This loss of TFF1-ir cells is better recognized on the enlarged images (B2). Scale bars: 1 mm (overview), 500 µm (magnification).

Given the significant co-localization of TFF1-ir cells with TH-ir cells we investigated whether TFF1-ir cells in the SN were projection neurons. For that purpose, the retrograde tracer Fluorogold (FG) was injected into the right striatum of adult rats. Two weeks after injection FG was detected in numerous cells in the SN and a subpopulation of TFF1-ir cells was found to co-express FG (Figure 7). As expected, a high number of TH-ir cells co-expressed FG, however, not all TH-ir cells were found to be FG labelled. To further verify the notion that TFF1-ir cells may represent dopaminergic projection neurons we performed immunohistochemical analyses for TFF1 in an animal model of Parkinson’s disease (PD). As expected, the SNc was markedly depleted of TH-ir neurons on the lesioned side, four weeks after a striatal 6-OHDA administration, while TH-ir cells remained intact in the VTA (Figure 8 A1-2). In agreement with the outcome of the FG labelling experiments, we also found decreased numbers of TFF1-ir cells on the lesioned hemisphere, compared to the non-lesioned hemisphere, showing that a significant subpopulation of the TFF1-ir cells project to the striatum (Figure 8 B1-2).

## Discussion

The present study shows for the first time that trefoil factor 1 (TFF1) is markedly expressed in the developing and adult rat ventral mesencephalon, which is of particular interest in relation to Parkinson’s disease (PD). TFF1 was detected in a subpopulation of dopaminergic neurons within substantia nigra pars compacta (SNc) from postnatal day 7 (P7) to adulthood. This subgroup of TFF1-immunoreactive (-ir) neurons project to the striatum as shown by retrograde labelling, as well as by loss of TFF1-ir cells in the SNc after unilateral, intrastriatal 6-hydroxydopamine (6-OHDA) injection.

### TFF1 expression in adult rat midbrain neurons

TFF1-ir cells were found throughout the adult rat mesencephalon, but with a distinct distribution pattern, with the most pronounced expression in the SN, ventral tegmental area (VTA) and in the periaqueductal grey matter and the Edinger-Westphal nucleus. These findings suggest that TFF1 might be involved in a number of complex functions in the midbrain as the periaqueductal grey plays a role in the descending modulation of pain and in defensive behaviour [36]. The Edinger-Westphal nucleus, also known as the accessory oculomotor nucleus, contains parasymphatic neurons that innervate the eye, specifically those that contract the pupil and adjust the lens [37].

With focus on the ventral mesencephalon, we found that TFF1 was exclusively expressed in neurons and not in astrocytes. This was both based on the morphological appearance of the cells, co-localization with NeuN and the lack of co-localization with an astroglial marker. Notably, a recent study has reported that NeuN expression levels in the rat SN are highly variable, with significant understaining, and that a substantial number of dopaminergic neurons showed little or no NeuN expression [38]. This is in line with our observation that a number of TFF-ir cells did not co-localize with NeuN, and therefore does not exclude the possibility that these cells are neurons.

From the few studies of TFF1 in the CNS, astrocytes have been reported to represent the major site of hippocampal TFF1 mRNA synthesis [27–29]. However, these studies, performed by a single research group, investigated mRNA levels and no studies have followed showing protein expression levels. Nevertheless, such an observation may hint to the hypothesis of a potential retrograde transport of TFF1 from astrocytes in the striatum to nigrostriatal dopaminergic neurons. Our finding of a lack of co-expression for TFF1 and the astroglial marker GFAP in the striatum, however, does not support this hypothesis (see Figure S5).

Interestingly, in this context, the recent report by Baydyuk and co-workers demonstrated that brain-derived neurotrophic factor and neurotrophin-3 are anterogradely transported from midbrain dopaminergic neurons to support the survival of immature medium-sized spiny neurons in the developing mouse striatum. They found that striatal neuron numbers increased between P0 and P21 both in control and neurotrophin-3 knockout mice, however, the number was significantly reduced in the neurotrophin-3 knockout mice [39]. Hence, it is tempting to speculate that TFF1 expressing dopaminergic neurons may provide throphic support to their target cells. Such an assumption, however, requires additional experiments and remains speculative at the moment.

Our analyses revealed that TFF1 expression was predominantly found in SNc and SN pars lateralis (SNl) and to a lesser extent in the VTA, whereas TFF1 staining was absent in the SN pars reticulata (SNr). Thus, the staining pattern resembled that of dopaminergic neurons in these areas. In line with this notion, double-immunofluorescence analyses revealed that a high proportion of the TFF1-ir cells co-localized with tyrosine hydroxylase (TH). In contrast, however, only a subgroup of TH-ir neurons expressed TFF1. Further evidence for the existence of dopaminergic TFF1-ir cells in the SN was provided by FG tracing and intrastriatal injection of the neurotoxin 6-OHDA. We demonstrated that a subpopulation of TFF1-ir cells showed co-localization with both TH, and FG injected into the striatum and retrogradely transported to the SNc. This observation indicates that these cells are dopaminergic projection neurons. As expected, a high number of TH-ir cells in the SNc co-expressed FG, however, not all were labelled. Since we only applied the FG injection to one restricted site in the striatum a limited number of dopaminergic neurons were targeted. Nevertheless, proof that a subpopulation of TFF1-ir cells are indeed projecting to the striatum was further substantiated by a marked loss of TFF1-ir cells in SNc of 6-OHDA-lesioned hemiparkinsonian rats. However, a more detailed study is necessary to address co-localization of TH and TFF in the SNc of the lesioned rats. Hence, we can at present only speculate on the total number of nigral TFF-ir cells being dopaminergic projection neurons.

The finding that nigral TFF1 expression was found in calretinin (CR)-ir cells and in some calbindin (CB)-ir cells is indicative of several subpopulations of dopaminergic TFF-ir cells. As described previously, the midbrain dopaminergic neurons can be separated into a dorsal and a ventral tier. The dorsal tier includes CB-ir cells located in the dorsal part of both SN and VTA, and some cells of the A8 cell group. The ventral tier comprises a sheet of more densely packed cells located in the ventral parts of SN and VTA, which are CB-negative and are mostly positive for the ion channel protein Girk2 [40,41]. The ventral tier neurons project exclusively to the striatum where they mainly innervate the patch compartments. The CB-ir neurons of the dorsal tier constitute a more mixed population, comprising cells that project not only to limbic and cortical areas, but also the matrix compartment of the striatum [42,43]. However, future analysis needs to clarify the phenotype of the TFF1-ir projection neurons.

Taken together, TFF1-ir cells represent a subset of dopaminergic neurons in the rat ventral mesencephalon, but further evaluation is needed to clarify the specific functions and characteristics of this subtype in more detail.

### TH and TFF1 co-expression during development

Additional analysis of the developmental profile of TFF1 expression in the ventral mesencephalon showed that TFF1 expression was nearly absent in newborn rats (postnatal (P) day 0), whereas numerous TFF1-ir cells were detected in the SN and VTA at later developmental stages, i.e. at P7, P14 and P21. Dopaminergic neurons in the ventral mesencephalon are generated during a period extending from embryonic (E) day 12 to E16 in rats with a peak of genesis at E13 [44]. Naturally occurring cell death in the midbrain dopaminergic system starts shortly before birth in rats with an initial peak at P2 and a second peak around P14 [45]. During these two periods, TH-ir cells die by apoptosis, however, little is known about the specific cell death mechanism [45,46]. Thus, the proliferation and generation of dopaminergic neurons have stopped before substantial TFF1 expression appears within the ventral mesencephalon, hence it seems that TFF1 does not play a role in the initial generation of dopaminergic neurons. We hypothesize that TFF1 could be involved in differentiation and/or survival of dopaminergic neurons. Quantification of co-expressing cells showed that the percentage of TH-ir cells expressing TFF1 in the SN was significantly higher in P7 and P14 rats compared to P21, indicating that a population of the TH/TFF1 co-expressing cells down-regulate TFF1 or die during development. However, the percentage of TFF1-ir cells co-expressing TH (around 85%) was found to be unchanged. Further investigations are needed to clarify the phenotype of TFF1 expressing cells and the possible functions of TFF1 during the development and maintenance of dopaminergic neurons.

## Conclusion

In conclusion, TFF1 displays a distinct protein expression pattern in the developing and adult rat midbrain with marked expression in a significant subset of dopaminergic projection neurons in SNc. TFF1 may as such represent a very useful supplementary marker, but its functional role and whether it plays a role in the pathophysiology of PD, however, remains to be elucidated.

## Supporting Information

Figure S1 **Photomicrographs of sections from the ventral mesencephalon and stomach of adult rats immunostained for trefoil factor 1 (TFF1).** Control staining was performed by omitting the primary antibody (-1° ab). Distinct TFF1-immunoreactive (-ir) cells were found in the adult rat ventral mesencephalon containing the substantia nigra (SN) as well as in the gastric tissue (left panel). Moreover, no TFF1 staining was found in rat SN or gastric tissue when omitting the primary antibody (right panel). Scale bar: 50 µm.
(TIF)

Figure S2 **Photomicrographs of adult rat brain sections at the level of the mesencephalon immunostained using different anti-trefoil factor 1 (TFF1) antibody concentrations (Novocastra), i.e. 1:250 (A), 1:500 (B), 1:2000 (C).** A1, B1 and C1 show a higher magnification of the substantia nigra pars lateralis. A2, B2, C2 show a higher magnification of the substantia nigra pars compacta. Note the different staining pattern of the TFF-ir cells and the difference in background staining for the different antibody dilutions used. Scale bars: 1mm (top row); 100 µm (lower rows).
(TIF)

Figure S3 **Photomicrographs of adult rat brain sections immunostained using mouse monoclonal anti-trefoil factor 1 (TFF1) antibody (Zymed Lab; 1:50; A, C, E) and rabbit polyclonal anti-TFF1 antibody (Novocastra; 1:1000; B, D, E).** Note the similar staining patterns of TFF1-ir cells seen for both antibodies at the level of the substantia nigra pars compacta (SNc), the periaqueductal grey matter (PAG), including the Edinger-Westphal nucleus (*) (C, D) as well as at the level of the subfornical organ (SFO) (E, F). Scale bar: 500 µm.
(TIF)

Figure S4 **Photomicrographs of sections through adult rat ventral mesencephalon at 4 weeks after unilateral 6-hydroxydopamine (6-OHDA) lesion of the nigrostriatal pathway immunostained using mouse monoclonal anti-trefoil factor 1 (TFF1) antibody (Zymed Lab.; 1:50).** The lesion resulted in a distinct loss of tyrosine hydroxylase (TH)-ir neurons in right SN as compared to the contralateral, unlesioned control side (see Figure 8). Similarly, a reduction of TFF1-ir cells was detected on the lesioned side (B) as compared to the intact control side (A). This loss of TFF1-ir cells is better recognized on the enlarged images (C, D). Scale bars: 1 mm (overview), 400 µm (magnification).
(TIF)

Figure S5 **Representative photomicrographs showing a double-immunofluorescence staining for trefoil factor 1 (TFF1) and the astroglial marker glial fibrillary acidic protein (GFAP) in the dorsal striatum of adult rats.** TFF1-ir cells (arrows) did not co-localize with GFAP (arrowheads). Scale bar: 50 µm.
(TIF)
