# Supplementary figures and images for: Expression of Trefoil Factor 1 in the Developing and Adult Rat Ventral Mesencephalon
Source: PLoS One. 2013 Oct 7;8(10):e76592. doi: 10.1371/journal.pone.0076592 (PMC3792045; doi:10.1371/journal.pone.0076592)

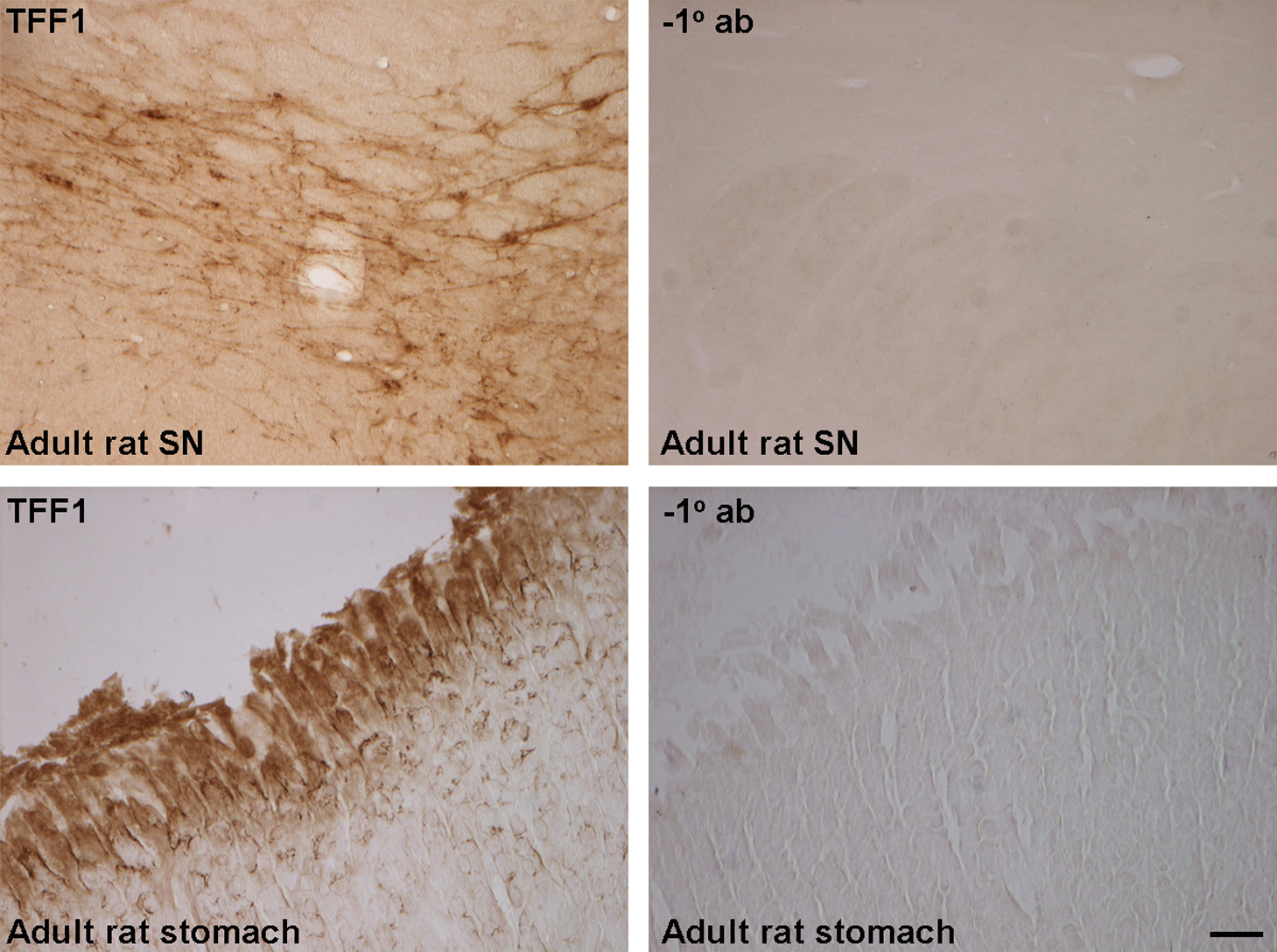

Supplement: Figure S1 — Photomicrographs of sections from the ventral mesencephalon and stomach of adult rats immunostained for trefoil factor 1 (TFF1). Control staining was performed by omitting the primary antibody (-1° ab). Distinct TFF1-immunoreactive (-ir) cells were found in the adult rat ventral mesencephalon containing the substantia nigra (SN) as well as in the gastric tissue (left panel). Moreover, no TFF1 staining was found in rat SN or gastric tissue when omitting the primary antibody (right panel). Scale bar: 50 µm. (TIF) [file pone.0076592.s001.tif]

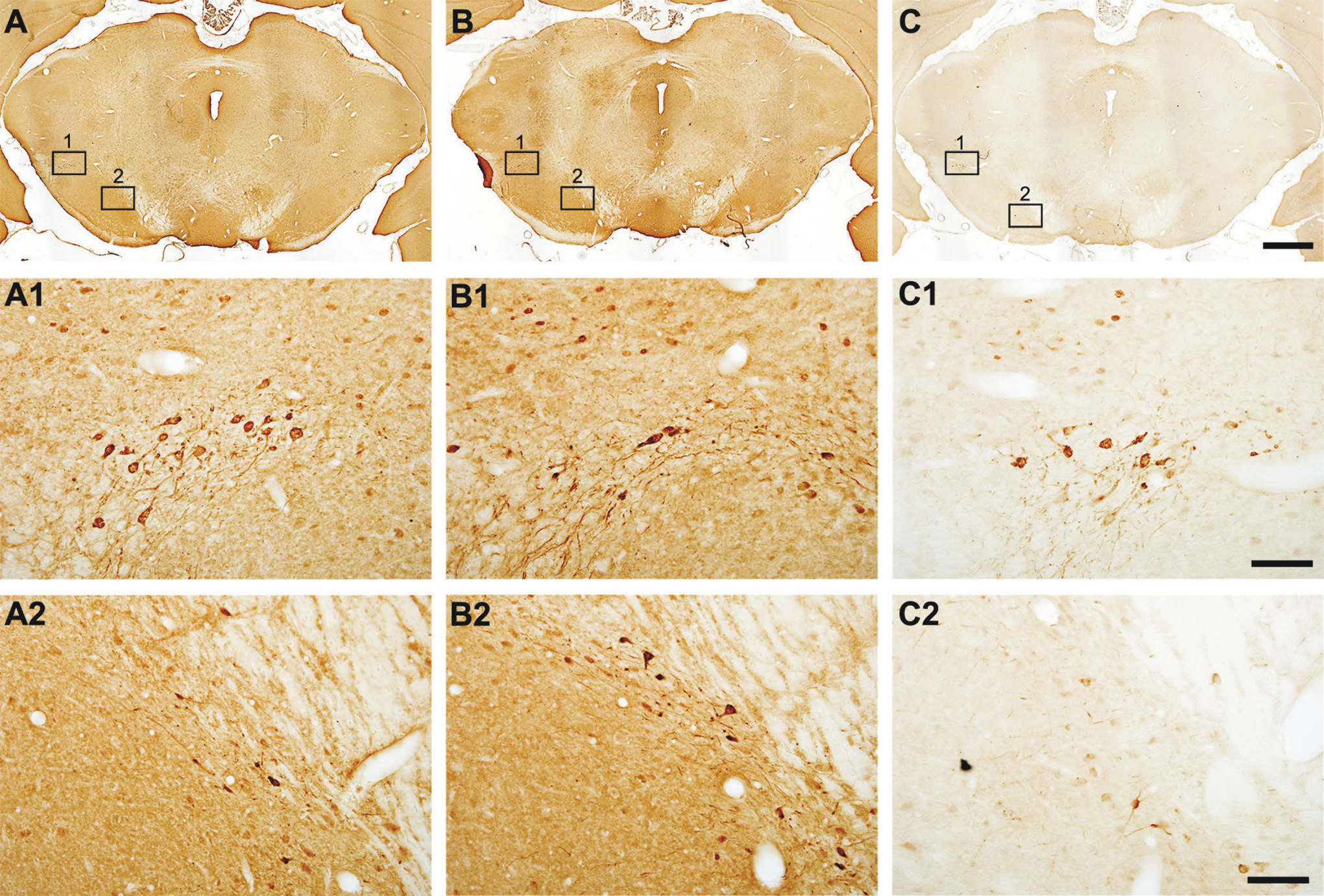

Supplement: Figure S2 — Photomicrographs of adult rat brain sections at the level of the mesencephalon immunostained using different anti-trefoil factor 1 (TFF1) antibody concentrations (Novocastra), i.e. 1:250 (A), 1:500 (B), 1:2000 (C). A1, B1 and C1 show a higher magnification of the substantia nigra pars lateralis. A2, B2, C2 show a higher magnification of the substantia nigra pars compacta. Note the different staining pattern of the TFF-ir cells and the difference in background staining for the different antibody dilutions used. Scale bars: 1mm (top row); 100 µm (lower rows). (TIF) [file pone.0076592.s002.tif]

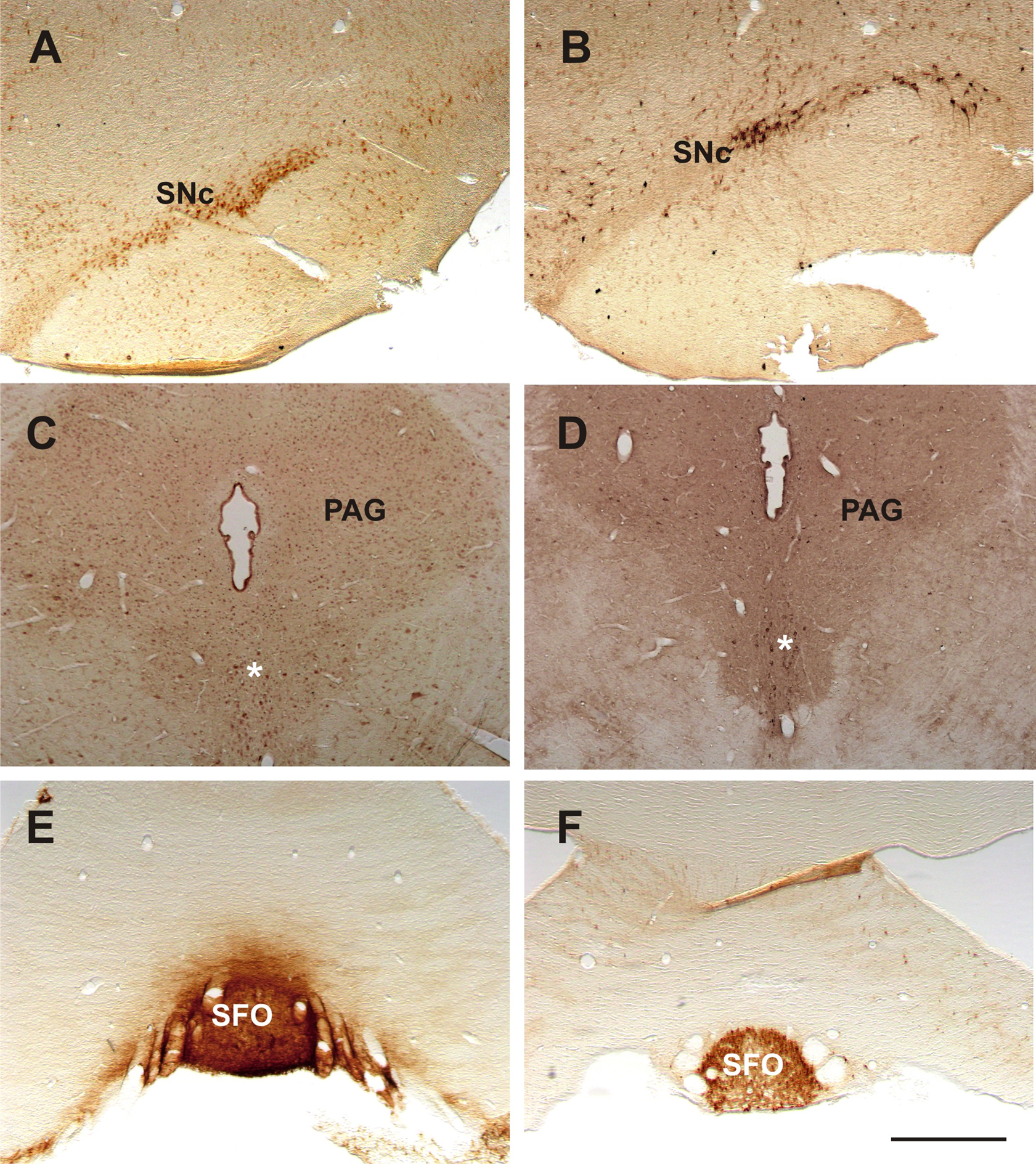

Supplement: Figure S3 — Photomicrographs of adult rat brain sections immunostained using mouse monoclonal anti-trefoil factor 1 (TFF1) antibody (Zymed Lab; 1:50; A, C, E) and rabbit polyclonal anti-TFF1 antibody (Novocastra; 1:1000; B, D, E). Note the similar staining patterns of TFF1-ir cells seen for both antibodies at the level of the substantia nigra pars compacta (SNc), the periaqueductal grey matter (PAG), including the Edinger-Westphal nucleus (*) (C, D) as well as at the level of the subfornical organ (SFO) (E, F). Scale bar: 500 µm. (TIF) [file pone.0076592.s003.tif]

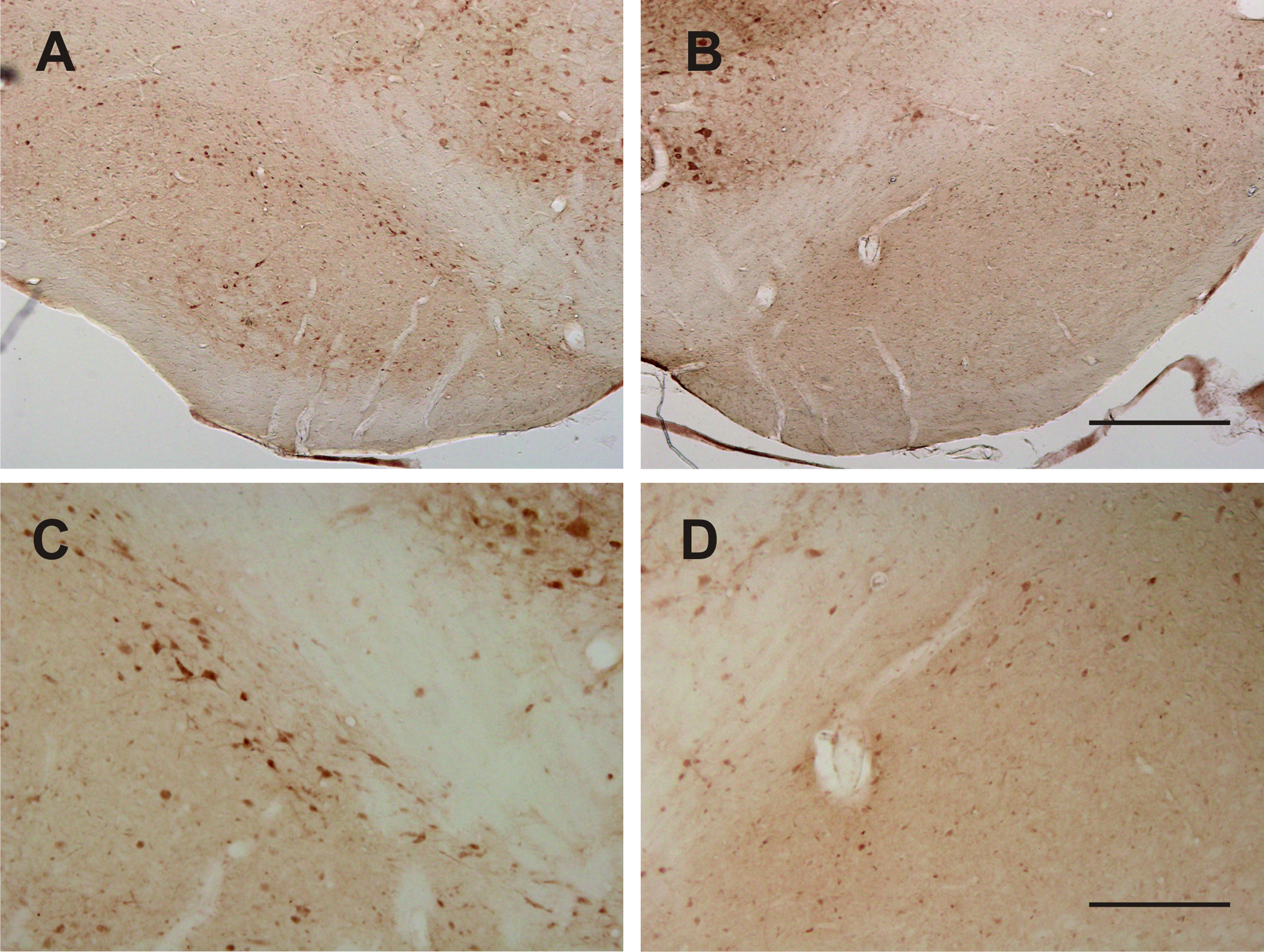

Supplement: Figure S4 — Photomicrographs of sections through adult rat ventral mesencephalon at 4 weeks after unilateral 6-hydroxydopamine (6-OHDA) lesion of the nigrostriatal pathway immunostained using mouse monoclonal anti-trefoil factor 1 (TFF1) antibody (Zymed Lab.; 1:50). The lesion resulted in a distinct loss of tyrosine hydroxylase (TH)-ir neurons in right SN as compared to the contralateral, unlesioned control side (see Figure 8). Similarly, a reduction of TFF1-ir cells was detected on the lesioned side (B) as compared to the intact control side (A). This loss of TFF1-ir cells is better recognized on the enlarged images (C, D). Scale bars: 1 mm (overview), 400 µm (magnification). (TIF) [file pone.0076592.s004.tif]

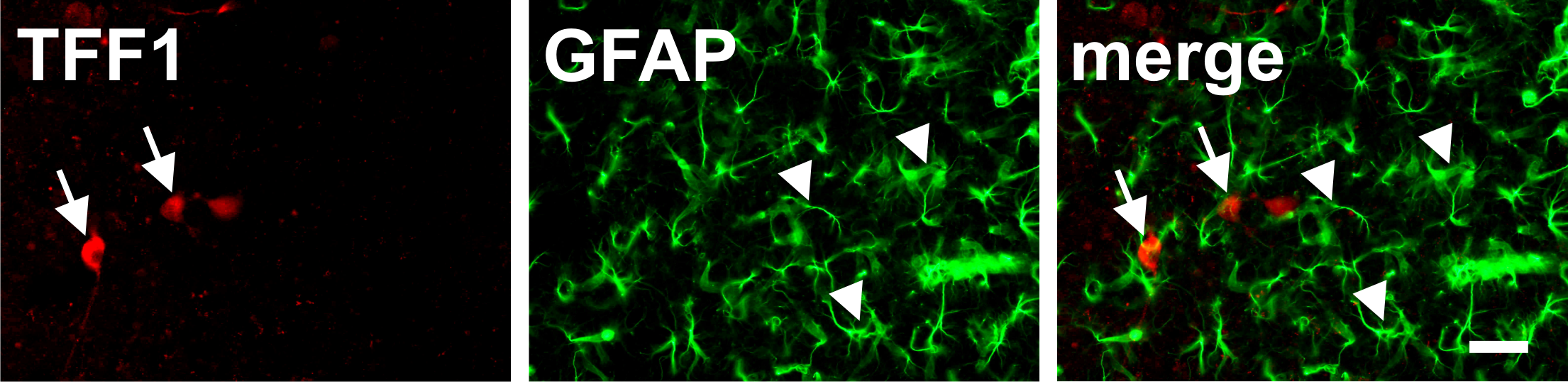

Supplement: Figure S5 — Representative photomicrographs showing a double-immunofluorescence staining for trefoil factor 1 (TFF1) and the astroglial marker glial fibrillary acidic protein (GFAP) in the dorsal striatum of adult rats. TFF1-ir cells (arrows) did not co-localize with GFAP (arrowheads). Scale bar: 50 µm. (TIF) [file pone.0076592.s005.tif]
